# Maintenance Cognitive Stimulation Therapy (CST) in practice: study protocol for a randomized controlled trial

**DOI:** 10.1186/1745-6215-13-91

**Published:** 2012-06-26

**Authors:** Amy Streater, Aimee Spector, Elisa Aguirre, Juanita Hoe, Zoe Hoare, Robert Woods, Ian Russell, Martin Orrell

**Affiliations:** 1 Unit of Mental Health Sciences, University College London, Charles Bell House, 67-73 Riding House Street, London, UK; 2 Department of Clinical, Educational and Health Psychology, University College London, 1-19 Torrington Place, London, UK; 3 IMSCaR, room 105, Y Wern, Bangor University, Bangor, UK; 4 DSDC Wales, 45 College Road, Bangor University, Bangor, UK; 5 Bangor University, Brigantia Building, Lon Penrallt, Bangor, UK; 6 North East London Foundation Trust, Goodmayes Hospital, Ilford, London, UK

**Keywords:** Cognitive stimulation, Dementia, Staff, Training

## Abstract

**Background:**

Cognitive Stimulation Therapy (CST) is a psychosocial evidence-based group intervention for people with dementia recommended by the UK NICE guidelines. In clinical trials, CST has been shown to improve cognition and quality of life, but little is known about the best way of ensuring implementation of CST in practice settings. A recent pilot study found that a third of people who attend CST training go on to run CST in practice, but staff identified a lack of support as a key reason for the lack of implementation.

**Methods/design:**

There are three projects in this study: The first is a pragmatic multi-centre, randomised controlled trial (RCT) of staff training, comparing CST training and outreach support with CST training only; the second, the monitoring and outreach trial, is a phase IV trial that evaluates implementation of CST in practice by staff members who have previously had the CST manual or attended training. Centres will be randomised to receive outreach support. The primary outcome measure for both of these trials is the number of CST sessions run for people with dementia. Secondary outcomes include the number of attenders at sessions, job satisfaction, dementia knowledge and attitudes, competency, barriers to change, approach to learning and a controllability of beliefs and the level of adherence. Focus groups will assess staff members’ perceptions of running CST groups and receiving outreach support. The third study involves monitoring centres running groups in their usual practice and looking at basic outcomes of cognition and quality of life for the person with dementia.

**Discussion:**

These studies assess the effects of outreach support on putting CST into practice and running groups effectively in a variety of care settings with people with dementia; evaluate the effectiveness of CST in standard clinical practice; and identify key factors promoting or impeding the successful running of groups.

**Trial registration:**

Clinical trial ISRCTN28793457.

## Background

The worldwide population is rapidly aging [1], resulting in increased numbers of people with dementia [2]. The increase in demand for dementia-related services has long been anticipated. However, the planning and provision of services for people with dementia appear to be failing to meet increasing requirements [3]. Two thirds of people living in care homes have dementia, which in turn leads to an increasing level of dependency that can be attributed to a lack of stimulation, increased behavioural problems and the use of anti-psychotic medication [4]. There is a need to provide services in the community but also to improve the availability of psychosocial interventions.

CST is an evidence-based group programme for people with mild to moderate dementia [5]. A review of reality orientation (RO) [6] helped to develop CST as a brief psychosocial group intervention, focussing on implicit information processing. The development of CST has followed the Medical Research Council (MRC) guidelines for the development and evaluation of complex interventions [7]. Cognitive stimulation leads to benefits in cognition and quality of life in the person with dementia [8], is shown to be cost effective and compares favourably with cholinesterase inhibitors for Alzheimer’s disease [5,9]. Currently cognitive stimulation is the only non-pharmacological intervention to improve cognition recommended in the NICE-SCIE Guidelines for Dementia [10], which recommend that all people with mild to moderate dementia should be ‘given the opportunity to participate in a structured group cognitive stimulation programme’.

CST is a twice-weekly, 7-week programme of stimulating 45-min group activities for people with dementia. A pilot study [11] of CST with an additional 16 weeks of once-weekly maintenance CST (MCST) sessions found a continued improvement in cognitive function for the people with dementia receiving maintenance CST. A large-scale RCT of maintenance CST (24 weeks of once-weekly sessions) versus CST only [12] has recently been completed as part of the SHIELD (Support at Home Interventions to Enhance Life in Dementia) project. The programme includes 7 weeks of CST and a further 24 weeks of maintenance CST sessions.

In recent years there has been an increase in the provision of CST, with around a third of the community mental health teams in the UK reporting using it [13]. This has been facilitated by the publication of the CST ‘Making a difference’ manual and the maintenance CST manual ‘Making a difference 2’ with a CST staff-training DVD [14,15]. However, there has been little research on the long-term implementation and evaluation of CST in practice.

A recent study found that after attending a CST training course, one third of the staff went on to implement CST groups in practice [16]. All staff members felt skilled enough to run the groups; however, they identified the need for management support, regular supervision, supervision from a specialist, online forums and additional training as useful in starting and running groups. Previous research into CST has reached phase III of the MRC framework for complex interventions [7]. This research aims to study the long-term implementation of CST within two linked trials and conduct a phase IV trial that will evaluate the effects of training, and the effects of outreach support and the long-term effects of CST in practice, and will help to understand the barriers and facilitators related to implementing CST in practice.

## Methods

### Design

This study includes three projects. The training and outreach trial and the monitoring and outreach trial differ in how staff members are recruited and in their previous exposure to training in CST. The observational study focuses on the effects of CST on people with dementia in the practice context. The three projects together will provide evidence on the most effective way of facilitating the implementation, uptake and effectiveness of the approach in a clinical context (see Table 1).

**Table 1:** Project overview

**Title**	**Training and outreach trial**	**Monitoring and outreach trial**	**Observational study**
**Aim**	To assess the effectiveness of staff training and outreach support	To assess the implementation in practice of CST and outreach support	To assess the effectiveness of CST in practice
**Participants**	Dementia care staff	Dementia care staff	People with dementia
	No previous CST experience or training	Previously received CST manual/training	
**Number**	120	120	100
**Resources**	CST manual maintenance CST manual DVD	CST manual maintenance CST manual DVD	CST manual maintenance CST manual DVD
**Training**	Yes	Variable	Variable
**Outreach**	50%	50%	No
**Assessment timeframe**	Baseline, 6 and 12 months	Baseline, 6 and 12 months	Before and after CST (0, 7 or 0, 14 weeks) and maintenance CST (31 or 38 weeks)

### Training and outreach trial (TROU)

**Figure 1:**
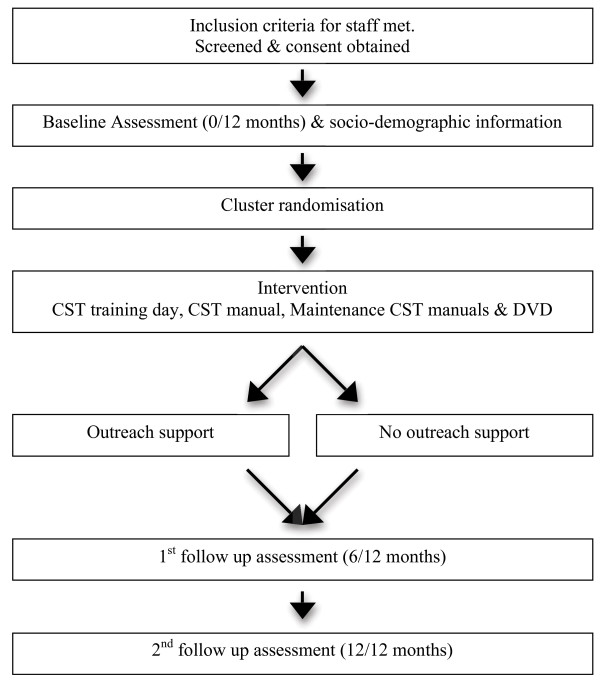
Flow diagram of training and outreach trial and assessment schedule.

The design is a pragmatic, multi-centre, single-blind, two treatment arm, randomised controlled trial. All participants receive the training package as treatment as usual (TAU) consisting of the 1-day CST training, training DVD, CST manual and maintenance CST manual. Participants in the intervention group also receive outreach support (local coordinator, email support and online forum). The sample is dementia care staff from specialist and non-specialist dementia care settings. The staff are cluster randomised according to place of work, before the training day, to receive outreach support or not. Each staff member completes three questionnaires, the first before the training day and at 6 and 12 months thereafter (Figure 1).

### Recruitment

Recruitment to the trial includes staff from care homes, day centres and NHS Trusts from various locations across the UK. The trial is advertised through the Journal of Dementia Care, and the National Care forum and referrals are made through the commercial CST training day. The research team is then able to assess their eligibility for participating in the research. Follow-ups will also be made with centres that have expressed a previous interest in CST or attending a CST training day.

### Participants

Staff members are screened to ensure they meet the inclusion criteria: (1) adequate written and spoken English, (2) able to complete online assessments at three different time points, (3) have at least two other team members with whom to run groups, (4) agreement from their management to have 2 h set aside per week to run the CST groups and 1 h following on from this for the 24-week maintenance CST programme, and (5) are able to provide between five to eight people with mild to moderate dementia who are willing to participate and meet the inclusion criteria (described in the observational arm). A minimum of three staff members is recruited per centre for logistical reasons of being able to consistently run the groups. Up to 40 centres are needed to be able to recruit the 120 staff members required for the trial. Working on the premise of a 15% attrition rate, the sample size will provide sufficient numbers for the staff training outreach support/no outreach support to estimate effect size and the feasibility of the trial. The attrition rate is an estimation based on previous research conducted in CST [12].

### Training package

The CST and maintenance CST manual along with the DVD are distributed to all staff members participating in the trial. The CST ‘Making a difference’ manual describes an evidence-based programme of group activities providing stimulation for people with mild to moderate dementia based on the principles of person-centred care. The maintenance CST manual, ‘Making a difference 2’, follows on from the original programme and includes a 24-week structured programme of activities aimed at challenging yet empowering the person with dementia and comes with a staff-training DVD. The staff-training DVD comprises an introduction by Dr Aimee Spector as to what CST is, a table listing the order of the CST and maintenance CST sessions. and key principles. In addition to this, there is video footage of CST sessions with people with dementia for staff to observe and discuss. After each clip there are questions based on the key principles to encourage reflective learning and discussion. All staff members attend a CST training day. The training comprises the different perspectives of dementia, the main psychosocial approaches for dementia, the development and evaluation of CST, the session themes and examples of activities for sessions, key principles, planning of sessions and the setting up of a group, and reflective learning of issues that arise when implementing and running groups. The training uses learning methods such as role-play, small group exercise and use of the training DVD, and time is spent reflecting on the sessions and critically appraising how the session is run. Following on from the training day, it is advised that each session be run with two facilitators to enable them to jointly plan and reflect on the session, and complete the attendance and adherence forms together.

### Randomisation

Cluster randomisation occurs prior to the staff attending the CST training day to ensure that staff members from the same centre receive the same level of support. The allocation ratio for randomisation is 1:1, into either the intervention or TAU. Randomisation is managed by email to the North Wales Organization for Randomized Trials in Health (NWORTH), which is an accredited trials unit, specialising in pragmatic trials.

### Treatment as usual

Staff members within centres that are randomized to the control group deliver the CST as usual but without the additional outreach support. This can vary between centres and has the variability to change over time, but the training package offered to the intervention group will also be available to those in the control group. Therefore, the trial examines the additional effects of the outreach support.

### Intervention

A pilot study conducted by our team identified that outreach support should consist of (1) an online forum, (2) email support and (3) local supervision [16]. The online forum is an online discussion site. It is accessible by user name and password. The first time a person attempts to enter the site, an email is sent to a member of the research team for approval in order to ensure they have been randomised to the intervention group. The use of a login allows us to record the number of people accessing the service, and how many times. Staff members are able to write up a variety of messages ranging from comments on sessions, to questions and advice. A researcher, who has extensive experience of CST and experience of running groups, delivers the email support (Amy Streater) and the service is made available as much as is needed by the staff members. A person familiar with CST and experience in running groups delivers the local supervision. The centre identifies the relevant person; however, if this is not possible a member of the research team provides the support. The role of the local supervisor is to help with the setting up of the CST group and also the practical issues that the staff members encounter when attempting to run CST groups. The supervisor records all the support given.

### Primary outcome measure

The primary outcome measure is number of CST and maintenance CST sessions run in the centre by the follow-up at 31 weeks. This is recorded using the monitoring progress form located in the ‘Making a difference’ manual [14], which includes who was in attendance, level of interest, communication, enjoyment and mood, on a rating scale 1–5. This measure is being completed at the end of each session from baseline to 31 weeks (inclusive of maintenance CST), until the maintenance CST groups have been completed or until the groups have been discontinued.

### Secondary outcome measures

a) The level of adherence to the CST and maintenance CST programme is measured by a adherence list designed as part of the research. It is based on the 18 key principles as developed as part of the maintenance CST programme [15]. The responses are reviewed by a researcher to mark whether staff are adhering to the key principles as laid out in the ‘Making a difference 2’ manual. Any ambiguity of responses given by the participants will be discussed with another researcher until a consensus is reached as to whether they are adhering to the key principles.

b) Job satisfaction [17] is measured using the Minnesota Satisfaction Questionnaire (MSQ). It is made up of 100 questions and comprises 20 dimensions with five items per scale with a 5-point Likert rating scale. The measure has adequate internal reliability.

c) Staff members’ approach to dementia is measured using the Approaches to Dementia Questionnaire (ADQ) [18]. The ADQ has 19 statements about the person with dementia and the care they receive. The scale has high validity and good reliability using Cronbach’s *α* for its person-centeredness and hopefulness subscales [18].

d) Knowledge is measured using the Dementia Knowledge–20 (DK-20) [19]. There are 20 questions for which there are five possible answers. The scale has sufficient reliability and is administered at baseline and final follow-up only.

e) Perceived sense of competence is measured using the Sense of Competence in Dementia care–Staff questionnaire (SCIDS) [20]. It comprises 17 items categorised into four subscales: professionalism, building relationships, care challenges and sustaining personhood. The scale has good internal consistency.

f) Learning characteristics of staff are measured using the brief Learning Transfer System Inventory (LTSI) [16,21]. The constructs of the LTSI are validated using common factor analysis [22,23]. The brief form comprises of 16 questions that are categorized into four major groups: trainee characteristics, motivation, work environment and ability [22]. All the items use five-point Likert-type scales from 1, strongly disagree, to 5, strongly agree.

g) Barriers to change within the workplace are measured using the Barriers to Change Questionnaire (BARCQ) [24]. It comprises 19 questions focussing on: institutional constraints, support from colleagues, philosophical opposition, client dissatisfaction, interference and positive factors. It also allows the addition of any further comments.

h) The emotional and behavioural responses relating to challenging behaviour presented by the person with dementia are measured by the Controllability Beliefs Scale [25]. The scale has 15 items based on a 5-point scale. The height of the score establishes the belief of the staff member in relation to the level of control demonstrated by the person with dementia. The scale has good internal reliability.

i) Focus groups with staff and managers will be conducted in both the TROU and MONOU trial to obtain qualitative data with regards to people’s perception of running groups and outreach support. They will run in a variety of care settings, and follow a semi-structured interview schedule, using inductive thematic analysis to code and analyse the gathered data.

### Consent

Staff members give informed consent and it is made clear that they will be of no disadvantage if they choose not to participate further at any stage during the trial. Consent is also sought from a member of management in order to give staff the optimum chance of carrying out CST in their workplace. Ethical approval was granted June 2011 by East London REC 3.

### Blinding

Although staff members cannot be blinded to their allocation, all assessment data are completed online and independently of the research team. Once the staff member has completed the survey, an administrator on the SHIELD programme team assigns all staff members a code for identification purposes to maintain anonymity throughout the trial. The researcher administering the outreach support has the contact details of the staff members but is unaware of their individual code; hence they are blind in identifying the staff members. However, it is common for participants to inadvertently inform researchers of the strand of the trial they have been allocated to. To reduce the risk of this, once staff members are aware of their code it is emphasised that it is not to be discussed with any members of the research team. This reminder is included in the email sent by a member of the research team to complete the follow-up assessments.

### Monitoring and outreach trial (MONOU)

**Figure 2:**
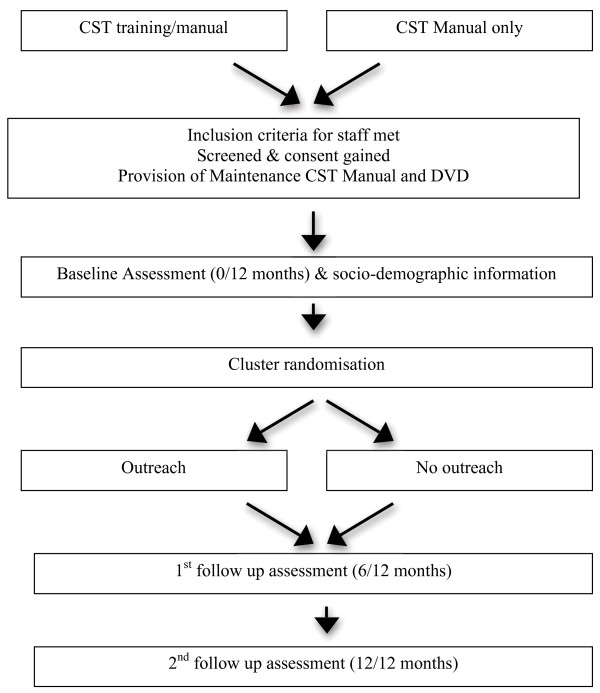
Flow diagram of the monitoring and outreach trial and assessment schedule.

The design is a pragmatic, multi-centre, single-blind phase IV trial. Participants are staff members from centres that have previously purchased the CST ‘Making a difference’ manual or attended a CST training course. The staff members are currently in possession of the CST manual or attended CST training. In addition to this, they all receive the maintenance CST manual and DVD. It is recorded whether participants have the manual only or manual and training. Centres are cluster randomised into outreach support or no outreach support (Figure 2). The time points for completing the questionnaire are the same as the training and outreach trial. However, they also complete a retrospective questionnaire on their use of CST in practice prior to the research.

### Recruitment

Recruitment of staff members who have purchased the manual is based on referrals by Hawker publications. A database of attendees generated from previously run CST training days allows us to approach people who have attended CST training. Staff members are then contacted to determine if they are interested in participating in the study. A CST poster advertises the research on the CST website (http://www.cstdementia.com), SHIELD (http://www.ucl.ac.uk/shield), and through the Journal of Dementia Care. The project is also a UKCRN portfolio-adopted study allowing NHS Trusts nationwide to be able to approach the research team in order to assess their eligibility for participating in the research.

### Participants

Participants are dementia care staff who have the CST manual or attended the CST training day, and are able to implement the CST programme once or twice weekly. The screening and inclusion criteria match those for the training and outreach study but a minimum of one staff member can be recruited per centre.

### Randomisation

Randomisation is identical to the TROU trial. Before randomisation it is recorded who has the manual or manual and training. At various time points during the recruitment of the participants the centres are divided into two clusters, taking into considerations the size of the centre and type of previous training (manual vs. manual and training). This matched pair of clusters is then independently cluster randomized to receive the intervention or TAU by remote email service to N-WORTH. Because of differing numbers per centre we will endeavour to keep similar numbers in the control and experimental group, with an allocation ratio for randomisation of 1:1, into either the intervention or TAU group.

### Treatment as usual

Sites that are randomised to the control group deliver the CST as usual. This can vary between centres and has the variability to change over time. The trial examines the additional effects of the outreach support and long-term effect in practice.

### Intervention

The outreach support options are identical to those in the training and monitoring study, with one difference: to emulate CST in practice, staff members identify the local supervisor, and if this is not possible, it is recorded accordingly. All the staff members randomised to receive outreach support are encouraged to use all the options available to them, but they are not compulsory. The staff member is monitored to measure their usage of the outreach support options.

### Primary outcome measure

The primary outcome is identical to the TROU trial. However, due to the nature of the recruitment, in that people are being recruited who have previously purchased the ‘Making a difference’ manual or attended CST training, it will be dependent on people’s interest in taking part in the research. As a minimum of one staff member can participate per centre; between 40 and 120 centres are required to recruit 120 staff members. This figure also accounts for a 15% attrition rate.

### Secondary outcome measures

The secondary outcome measures are identical to those in the TROU trial.

### Consent

Informed consent is gained from each staff member and a member of management, and is identical to the training and outreach trial. Ethical approval was granted June 2011 by East London REC 3.

### Blinding

The procedure for blinding is identical to the TROU trial.

### Analyses

#### *Training and outreach trial & Monitoring and outreach trial*

A combined analysis making use of the results from the training and outreach trial (120 participants) and the monitoring and outreach trial (120 participants) will be carried out. The primary outcome will be the mean number of CST sessions offered per centre assuming the intra-cluster correlation p to be 0.05. At the 6-month primary end point based on 240 staff members (120 outreach vs. 120 control) in the outreach group, the mean number of sessions offered is estimated to be 16 (SD 10) and in the control group the mean number of sessions offered is expected to be 12 (SD 10). Setting *p* at 0.05, power at 0.8, with the effect size at 0.4, then 200 participants would be required to demonstrate a difference between the groups.

Using all the participants recruited across both trials (TROU and MONO), a four-group comparison will also be made. This will compare training and outreach support, training and no outreach support, manual only and outreach, and manual only and no outreach support. This will determine if there are differences between these groups at 6 and 12 months.

### Observational study of CST in practice

**Figure 3:**
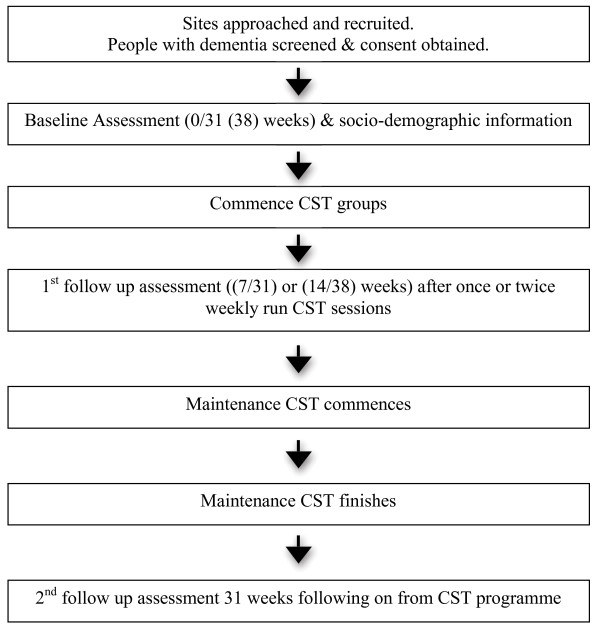
Flow diagram of observational study and assessment schedule.

The design is a multi-centre, longitudinal observational study with people with dementia. Sites that are currently running or in the process of setting up CST groups complete minimal outcome measures at three time points with people with dementia who are participating in the CST and maintenance CST programme (Figure 3). The measures are being completed before the group starts (baseline), 7 or 14 weeks depending on how they implement the CST programme (once or twice weekly), and after the maintenance CST at 31 or 38 weeks. The intervention is CST as routinely offered in the care setting. The aim of this study is to determine whether groups are running in practice and demonstrate the positive findings for cognition and quality of life of life for the person with dementia found in previous CST research [5,11].

### Recruitment

Centres that are running CST groups are approached, and the staff asked to complete measures in cognition and quality of life with the people with dementia taking part in the groups. The centre type, level of staff experience and training are recorded. The centres are given the maintenance CST manual and staff training DVD. Recruited participants have a confirmed diagnosis of mild to moderate dementia.

### Participants

Centres that are currently running or setting up CST groups are approached to participate in the observational study. Approximately 13 centres are needed to provide us with 100 people with dementia and experienced staff in dementia care. The person with dementia will have (1) a score of between 0.5 and 2 on the Clinical Dementia Rating scale [26] and (2) a diagnosis of dementia, (3) adequate spoken and written English, (4) the ability to participate in a ‘meaningful’ conversation and (5) remain in a group for 45 min. Participants also need (6) adequate eyesight and hearing, (7) to be able and willing to give informed consent, and (8) able to complete a cognition and quality of life measure at three intervals over a year. If five to eight people with dementia give informed consent to complete the minimal outcome measures, with a staff member or researcher, the centre is recruited in to the study and a letter explaining their participation in the research is sent to their GP.

### Training package

The staff members within the centres have the CST manual or previously attended CST training, which includes the CST manual. In addition the centre will receive the Maintenance CST manual and DVD.

### Randomisation

This is a naturalistic study of CST in practice so people with dementia who are about to start CST groups are approached and informed, and consent obtained. They are then assessed at three time points (0, 7 and 31 weeks or 0, 14 and 38 weeks) throughout their participation in the trial.

### Outcome measures

The primary and secondary outcome measures for people with dementia are completed at baseline (T0) prior to the CST programme starting, then post CST groups (T1) (at 7 or 14 weeks depending on whether groups are implemented once or twice weekly). The final follow-up will be completed 24 weeks after the maintenance CST has commenced (T2). Socio-demographic information is collected about the person with dementia including age, gender, race, diagnosis of dementia, type of diagnosis and medication. Medication will be recorded at each follow-up to mark any differences for the duration of their participation in the trial.

a) The primary outcome is cognition as measured by the Mini Mental State Examination (MMSE) [27]. The MMSE has a score of up to 30 points and is widely used as a brief indicator of level of cognitive impairment. It has good reliability and validity.

b) The secondary outcome measure is quality of life as measured by the Quality of Life-Alzheimer’s Disease (QoL-AD) [28]. The QoL-AD is a 13-item scale measuring different aspects of life. The scale totals 52 points and higher scores indicate better quality of life. It has good internal consistency, validity and reliability [28,29].

### Consent

It is expected that the participants are competent to provide informed consent to participate in the trial. The British Psychological Society guidance on evaluation of capacity will be adhered to. In addition, consent is an on-going process as opposed to a one-off decision and this will be continually monitored throughout the study. Mental Capacity Act [30] guidance will also be applied where relevant, such as when then a participant is no longer able to give informed consent. A person’s preference can be indicated by their initial willingness to participate in the trial. However, if any distress or discomfort is evident during the study the person will be withdrawn. Ethical approval was granted on June 2011 by East London REC 3.

### Blinding

Blinding is unnecessary for the staff members and researchers, as each person with dementia has the opportunity to participate in the CST and maintenance CST programme and the staff members are completing the assessment at each time point.

### Analyses

Analysis will be a pre-post analysis based on intention to treat, in that all collected data made available by the person with dementia will be included, regardless of whether they complete the programme or not. Imputation methods such as the last observation carried forward are of limited use in dementia, as there is the expectation of a gradual decline and for participants to be lost through illness or death. A linear regression model will be used where data are missing in order to predict the missing value and impute the total when possible. The sample size calculation accounts for the number of people expected to be available at the study end point. All participants are in receipt of the CST and maintenance CST programme. Analysis will take into account the evaluation at 24 weeks after CST as the primary end point. Secondary analysis will consider the effects immediately following the CST programme. Age, gender, cholinesterase inhibitor and baseline scores on the two scales being measured will be entered as covariates, together with ‘centre’ entered as a random factor.

### Ethical arrangements

#### *Risks and anticipated benefits for trial participants*

There appear to be no documented harmful side effects from participating in either CST training or in the running of the CST and maintenance CST programme, and no adverse reactions are apparent. People with dementia who have participated in previous CST groups consistently report benefits of feelings of validation, self-worth and overall enjoyment of the sessions [31]. Potential participants, both staff members and people with dementia, will be fully informed of the potential risks and benefits of the project. A reporting procedure is in place to ensure that serious adverse events (SAEs) are reported to the Chief Investigator. SAEs that are considered to be related and unexpected are reported to the Multicentre Research Ethics Committee and the trial Data Monitoring and Ethics Committee.

## Discussion

This project will pragmatically evaluate the effectiveness of staff training and outreach support by increasing the delivery of CST in practice by outreach support intervention both in new CST practitioners (TROU trial) and experienced CST practitioners (MONOU trial). The NICE-SCIE guidelines recommend CST, and this project aims to record the uptake and delivery of CST in dementia settings, to determine if it is easy to follow and replicable in practice. The MONOU trial provides a naturalistic evaluation of benefits of manual only versus manual and training in CST implementation, and both the TROU and MONOU trial will identify staff and situational factors that impede or facilitate CST implementation. One of the important advances in this study is to measure the adherence by specific questions developed in direct relation to the key principles that define CST as a therapy. It also allows the research to demonstrate, on a large scale, the knowledge, views and understanding, and approach of staff members to dementia in a variety of care settings nationwide with the secondary outcome measures. In relation to the observational study it provides an evaluation of the long-term cognitive and quality-of-life benefits of CST and maintenance CST in practice.

This study should provide definitive evidence of the effectiveness and feasibility of implementation of CST and maintenance CST in a variety of care settings. This study is likely to influence the availability, provisions and uptake of CST and maintenance CST in the UK and internationally, and may also impact on current evidence-based guidelines and policies relating to dementia care.

### Trial status

Ongoing

## Abbreviations

CST, cognitive stimulation therapy; UK, United Kingdom; TROU, training and outreach; MONOU, monitoring and outreach.

## Competing interests

AS, RTW and MO have co-authored a CST manual, and AS, RTW, MO, JH, EA and ASt co-authored the maintenance CST manual, the royalties from which are received by the Dementia Services Development Centre Wales. AS also runs the CST training course on a private basis.

## Authors’ contributions

MO developed the original concept of the trial, and AS, JH, ASt, EA, ITR and ZH developed the design and methodology; all authors reviewed and commented on drafts of the protocol and paper. All authors read and approved the final manuscript.
